# Nitrogen Amendment Stimulated Decomposition of Maize Straw-Derived Biochar in a Sandy Loam Soil: A Short-Term Study

**DOI:** 10.1371/journal.pone.0133131

**Published:** 2015-07-20

**Authors:** Weiwei Lu, Weixin Ding, Junhua Zhang, Huanjun Zhang, Jiafa Luo, Nanthi Bolan

**Affiliations:** 1 State Key Laboratory of Soil and Sustainable Agriculture, Institute of Soil Science, Chinese Academy of Sciences, Nanjing, China; 2 Land and Environment, AgResearch, Hamilton, New Zealand; 3 Centre for Environmental Risk Assessment and Remediation, University of South Australia, Adelaide, Australia; Chinese Academy of Sciences, CHINA

## Abstract

This study examined the effect of nitrogen (N) on biochar stability in relation to soil microbial community as well as biochar labile components using δ^13^C stable isotope technology. A sandy loam soil under a long-term rotation of C_3_ crops was amended with biochar produced from maize (a C_4 _plant) straw in absence (BC0) and presence (BCN) of N and monitored for dynamics of carbon dioxide (CO_2_) flux, phospholipid fatty acids (PLFAs) profile and dissolved organic carbon (DOC) content. N amendment significantly increased the decomposition of biochar during the first 5 days of incubation (*P* < 0.05), and the proportions of decomposed biochar carbon (C) were 2.30% and 3.28% in BC0 and BCN treatments, respectively, during 30 days of incubation. The magnitude of decomposed biochar C was significantly (*P* < 0.05) higher than DOC in biochar (1.75%) and part of relatively recalcitrant biochar C was mineralized in both treatments. N amendment increased soil PLFAs concentration at the beginning of incubation, indicating that microorganisms were N-limited in test soil. Furthermore, N amendment significantly (*P* < 0.05) increased the proportion of gram-positive (G^+^) bacteria and decreased that of fungi, while no noticeable changes were observed for gram-negative (G^−^) bacteria and actinobacteria at the early stage of incubation. Our results indicated that N amendment promoted more efficiently the proliferation of G^+^ bacteria and accelerated the decomposition of relatively recalcitrant biochar C, which in turn reduced the stability of maize straw-derived biochar in test soil.

## Introduction

Biochar is a solid material obtained from the thermochemical conversion of biomass in an oxygen-limited environment [1]. Researchers have shown that as a soil additive along with organic and inorganic fertilizers, biochar can significantly ameliorate soil properties and improve plant productivity and thus benefit agricultural ecosystems [2–3]. Because of their predominantly aromatic nature, biochars are considered to be recalcitrant in soils [4]. It was reported that mean residence time (MRT) of biochar was in the range of centuries to millennia [5]. However, Steinbeiss et al. [6] observed that biochar degraded much faster than previously predicated and MRT in their study was in the range of a few decades. Therefore, a large variation in biochar stability is observed among different studies and consequently the stability of biochar in soils warrants further investigation so that the environmental and economical consequences caused by its application to soils can be better evaluated.

Nitrogen (N) fertilization as a common agricultural management practice could induce changes in soil microbial community [7], which might in turn result in a shift in the functioning of soil biogeochemical cycles, including the cycling of soil organic carbon (SOC), and thus influence the biochar decomposition in soils. The effect of N amendment on biochar decomposition is still an open question with little available information [8]. Schulz and Glaser [9] found that combination of beech-wood-retort barbecue charcoal with fertilizer N led to accelerated biochar degradation on an infertile sandy soil while Santos et al. [8] found N amendment had no effects on the decomposition of wood biochar added to a granitic soil. Ding et al. [10] demonstrated that N application stimulated organic carbon (OC) degradation in a sandy loam soil of the North China Plain. Using fluorescence excitation emission spectrophotometry, Uchimiya et al. [11] reported that biochar extracts from different sources (almond shell, broiler litter, cottonseed hull and peacon shell) contained fulvic-like and humic-like structures, similar to those found in SOC. Therefore, given that biochar and SOC had a certain similarity in chemical structure, it was hypothesized that N might also enhance the decomposition of biochar in test soil. However, there is still no study to verify the hypothesis up to date.

Biochar has been shown to undergo both microbial and abiotic (e.g., photochemical oxidation) degradation [5], and recent studies suggested that the biologically mediated degradation of biochar might be the dominant pathway in soils [6]. Abiotic factors influencing biochar stability include physico-chemical characteristics of both biochar and soil, such as the proportion of labile OC and elemental compositions in biochar and soil texture and OC content [12–14]. Microbial utilization of biochar included respiration of biochar C as carbon dioxide (CO_2_) and incorporation of biochar into microbial biomass. Fungi, gram-positive (G^+^) bacteria and actinobacteria have been sequentially found to be able to directly utilize biochar C by Steinbeiss et al. [6], Santos et al. [8] and Watzinger et al. [15], respectively. The biochar decomposed by different species of microorganisms might be due to the differences in properties of biochar and soil used in different studies. Nevertheless, few reports have investigated the mechanisms involving the effect of N amendment on biochar decomposition up to date.

Therefore, the objectives of this study reported here were to: (i) evaluate the effect of N amendment on biochar decomposition and (ii) clarify the relationships between biochar decomposition and soil microbial community as well as biochar labile components as affected by N amendment in a sandy loam soil of the North China Plain. This study will benefit understandings of biochar stability and its interactions with N.

## Materials and Methods

### Biochar and soil samples

Biochar was produced from maize (a C_4_ plant) straw using a slow-pyrolysis process. Prior to pyrolysis, maize straws were oven-dried for 12 h at 80°C, and then transferred into the biochar reactor (China patent No. ZL200920232191.9). The reactor was heated by a step-wise procedure. The temperature was set at 200°C initially, and then elevated stepwise to 250, 300, 350, 400, 450 and 500°C. At each temperature step (except for 500°C), the process was maintained for 1.5 h. The whole process was flushed with nitrogen gas (N_2_) and terminated after about 13 h when there was no visible smoke emission from the gas vent. Selected properties of the biochar are shown in Table 1.

**Table 1:** Selected properties of the test biochar and soil.

Sample	pH	Total C (g C kg^–1^)	Inorganic C (g C kg^–1^)	Organic C-δ^13^C (‰)	DOC ^b^ (g C kg^–1^)	Total N (g N kg^–1^)	Total H (g H kg^–1^)	Ash (%)	Alkyl C (50–0 ppm) (%)	O/N-alkyl ^b^ C (95–50 ppm) (%)	Aryl C (165–95 ppm) (%)	Carbonyl C (220–165 ppm) (%)
**Biochar**	10.1 ^a^	639	3.51	-11.9	11.2	8.87	24.6	22.9	2.7	1.28	91.1	4.92
**Soil**	7.55	8.05	1.46	-24.5	0.56	0.76	ND ^c^	ND	ND	ND	ND	ND

^a^ The values denote means (*n* = 4).

^b^ DOC and O/N-alkyl denote dissolved organic carbon and oxygen/nitrogen-alkyl, respectively.

^c^ ND, not be determined.

Surface soil (0–20 cm) was collected from a field in the vicinity of Lugang town, Fengqiu county, Henan province, China (35°00’N, 114°24’E). The owner of the field gave permission to soil samplings. This field has been cultivated under a rotation of winter wheat (*Triticum aestivum* Linn.) and summer sweet potato (*Ipomoea batatas*), both C_3_ crops, for at least 50 years. The soil was developed from alluvial sediments of the Yellow River and classified as aquic inceptisol [16], with a sandy-loam texture (20% clay, 25% silt and 55% sand) (Table 1).

### Biochar and soil properties analyses

The contents of total C, N and hydrogen (H) in biochar were measured using the elemental analyzer (Vario MICRO, ELEMENTAR, Germany). The ash in biochar was determined gravimetrically after heating aliquots (~200 mg) of the sample to 760°C for 6 h in a ceramic crucible [17]. The pH of biochar and soil was determined with a glass electrode using solid-to-water ratios of 1:5 and 1:15, respectively. The higher ratio for biochar was due to its low density [18]. The δ^13^C values of biochar OC and SOC were measured using an isotope ratio mass spectrometer (Finnigan MAT 251, Thermo Electron, Germany) at Institute of Soil Science, Chinese Academy of Sciences. To determine the OC-δ^13^C values, the procedure of Midwood and Boutton [19] was followed for acid washing of the biochar and soil. Inorganic C contents in biochar and soil were determined by potentiometric titration [20]. Soil texture was analyzed by laser particle size analyzer (Beckman Coulter, LS, USA). The contents of OC and total N in soil were determined with wet oxidation-redox titration and micro-Kjeldahl methods, respectively [20].

To measure concentrations of dissolved organic carbon (DOC), biochar or soil was extracted with hot water according to the procedures of Chodak et al. [21]. Hot water extracts were obtained by boiling samples in deionized water for 1 h in a water bath pot. After cooling, the suspensions were centrifuged for 10 min at 2,000 g and filtered through 0.45 μm filters. DOC concentration of extracts was determined on a TOC analyzer (Multi N/C 3000, Jena, Germany). After the extracts were frozen-dried, the δ^13^C of DOC was analyzed by an isotope ratio mass spectrometer (IRMS 20–22, SerCon, Crewe, UK).

The quantitative direct-polarization magic angle-spinning (DPMAS) ^13^C nuclear magnetic resonance (NMR) spectral pattern of the biochar was obtained using a Bruker AV III 400-MHz spectrometer (Swiss) at the Nanjing University. The experiment was run in a Bruker 4 mm double-resonance MAS probe head. Biochar sample was packed into 4 mm diameter zirconia rotors with 5 mm long glass inserts at the bottom to constrain the sample to the space within the radio frequency coil. The measurement conditions were as follows: ^13^C resonance frequency 100.63 MHz, magic angle spinning frequency 14 kHz, acquisition time 10 ms, recycle delay time 12 s and the number of data points 2048. The ^13^C chemical shifts were externally referenced to the methylene resonance of Adamantane at 38.5 ppm. No correction was made for spinning sidebands. The functional groups of biochar OC were divided into alkyl, O/N-alkyl, aryl and carbonyl according to Solum et al. [22].

### Laboratory incubation

An incubation experiment was carried out over 30 days and included two treatments: biochar alone amendment (BC0) and combined amendment of biochar plus inorganic N to soil (BCN) with 21 replicates for each treatment. The soil and biochar samples were ground to pass through 2 and 0.25 mm sieves, respectively. A series of 250 mL Erlenmeyer flasks with 35 g of soil sample (on an oven-dried basis) were prepared. Biochar was added into designated flasks at the application rate of 0.5% of soil mass (on an oven-dried basis), which was equivalent to a field application rate of 15 t ha^−1^, and mixed well with soil. A solution of ammonium sulfate was added into designated flasks at the application rate of 100 mg N kg^–1^ (300 kg N ha^−1^) while the remaining flasks were added with the same volume of deionized water as control. This application rate was chosen according to the specific agricultural practice by local farmers [23]. Given soil moisture generally amounted to 80% water-filled pore space (WFPS) following basal fertilization with subsequent irrigation in field during maize growth season [24], soil moisture was adjusted to 80% WFPS by adding deionized water at the beginning of incubation. All flasks were covered with aluminum foils with needle-punctured holes to maintain aerobic conditions, and then incubated at 25°C in the dark. In order to maintain soil water content, deionized water was added with mini-pipette every other day by weighing flasks during the incubation.

Three replicate flasks from each treatment were used to measure soil CO_2_ efflux at 0.5 h and on days 1, 3, 5, 7, 9, 11 and 30 during incubation. To measure CO_2_ efflux, each flask was sealed using an airtight butyl rubber stopper perforated by centered Perspex tubes, vacuumed and flushed with fresh air using a multiport vacuum manifold. Additional 20 mL fresh air was then immediately injected into the flasks using a plastic syringe, completely mixed with headspace gas, and the same volume gas was sampled and injected into pre-evacuated vials as the zero time samples for analysis. The flasks were returned to the incubator, and another 20 mL headspace gas in the flasks was sampled after 4 h enclosure. After gas sampling, stoppers were removed from the flasks, and aluminum foils were reused to cover the flasks. CO_2_ concentration was measured using a gas chromatograph equipped with a thermal conductivity detector (TCD) operated at 60°C (Agilent 7890, Santa Clara, CA, USA). CO_2_ gas standards were supplied by National Research Center for Certified Reference Materials, Beijing, China. Values of δ^13^C in the emitted CO_2_ were measured using an isotope ratio mass spectrometer (Finnigan MAT 253, Thermo Electron, Germany) at Institute of Soil Science, Chinese Academy of Sciences. The left flasks were destructively used for soil samplings at 0.5 h and on days 1, 3, 7, 11, and 30, respectively, with 3 replicates for each treatment at each time to measure soil phospholipid fatty acids (PLFAs) profile and DOC content and δ^13^C.

### PLFAs analysis

Soil PLFAs was extracted following the procedures described by Brant et al. [25]. Briefly, 2.00 g soil was extracted with a solution of methanol, chloroform and phosphate buffer (with a ratio of 2:1:0.8). The soil extracts were centrifuged and the chloroform phases were collected. Phospholipids were separated from glycolipids and neutral lipids using silicic acid bonded solid-phase-extraction columns (Sep-Pak Silica, Waters Corp., USA) by sequential eluting with chloroform, acetone and methanol. Phospholipids were saponified and methylated to fatty acid methyl esters (FAME) at 37°C in water bath. Following that, FAME was dried under N_2_ at 25°C and finally dissolved in hexane containing a 19:0 FAME standard.

The concentration of PLFAs was analyzed with MIDI Sherlock Microbial Identification System (Newark, Delaware, USA). The identified PLFAs were assigned to five main groups of microorganisms, i.e. bacteria in general, gram-negative (G^−^) and G^+^ bacteria, fungi and actinobacteria. The PLFAs 14:0, 16:0, 17:0 and 18:0 were used as biomarkers for general bacteria; 14:1ω5c, i15:1G, i16:1H, 16:1ω9c, 16:1ω5c, 17:1ω8c, cy17:0, 16:1 2OH, 18:1ω5c, 11Me 18:1ω7c and cy19:0ω8c for G^−^bacteria; i14:0, i15:0, a15:0, i16:0, a16:0, i17:0 and a17:0 for G^+^ bacteria; 18:3ω6c (6, 9, 12) and 18:1ω9c for fungi; and 10Me17:0 and 10Me18:0 for actinobacteria [26]. Proportions of various microbial groups calculated by the ratio of PLFAs concentration assigned to specific microbial group to that of all microorganisms were used to estimate soil microbial community structure.

### Calculations

Carbon dioxide efflux derived from biochar (*F*_b_, mg C kg^–1^ h^–1^) was calculated as follows [18]:

$$F_{\text{b}}\text{ = }f_{\text{b}}\;\times \;F_{\text{total}}$$ (1)

where *F*_total_ is total CO_2_ efflux in soil-biochar system (mg C kg^−1^ h^−1^), and *f*_b_ is the ratio of CO_2_ efflux from biochar to total CO_2_ efflux and was calculated based on a two-component isotopic mixing model as follows [27]:

$$f_{\text{b}}\text{ = }\left(\delta \text{ - }\delta_{\text{s}}\right)\text{ / }\left(\delta_{\text{b}}\text{ - }\delta_{\text{s}}\right)$$ (2)

where *δ*_s_ is the δ^13^C of native SOC (‰), *δ*_b_ is the δ^13^C of OC in biochar (‰), and *δ* is the δ^13^C of CO_2_ emitted from biochar-amended soils (‰) and was calculated based on mass balance as follows [28]:

$$\delta \;=\;\left(\delta_{2}\;\times C_{2}-\delta_{1}\;\times C_{1}\right)/\left(C_{2}-C_{1}\right)$$ (3)

where *δ*_1_ and *δ*_2_ are the δ^13^C of CO_2_ sampled at zero time and 4 h after flask enclosure (‰), respectively, and *C*_1_ and *C*_2_ are the concentrations of CO_2_ in gases sampled at zero time and 4 h after flask enclosure (μL L^–1^), respectively.

Mean cumulative CO_2_ emission (*E*_CO2_, mg C kg^–1^) from biochar was calculated by summing the products of the averaged two neighboring fluxes, multiplied by their interval time during the incubation as follows:

$$E_{CO_{2}}=\overset{n}{\underset{i=1}{\sum^{}}}(F_{i+1}+F_{i})/2\times (t_{i+1}-t_{i})$$ (4)

where *F*_*i*_ and *F*_i+1_ are CO_2_ fluxes at the *i* ^th^ and (*i*+1)^th^ gas samplings, respectively, the term (*t*_i+1_ –*t*_*i*_) represents the interval between the *i* ^th^ and (*i*+1)^th^ gas samplings (h), and *n* is the total times of gas sampling. The decomposition proportion (*DP*, %) of added biochar C during incubation was calculated as follows:

$$DP=E_{CO_{2}}/BC\times \;100$$ (5)

where *BC* is the amount of biochar C in soils (mg C kg^–1^).

The amount of DOC derived from biochar (*DOC*_b_, mg C kg^–1^) in soils was calculated:

$$DOC_{\text{b}}\text{ = }f_{DOC-b}\;\times \;DOC_{\text{total}}$$ (6)

where *DOC*_total_ is the total DOC content in soils amended with biochar (mg C kg^−1^), and *f*_DOC-b_ is the ratio of DOC from biochar to total DOC and was calculated with a similar method with *f*_b_ by replacing δ^13^C of CO_2_ with that of DOC.

### Statistical analyses

The unit mg C kg^–1^ referred to mg C kg^–1^ soil and all data were expressed on the basis of oven-dried soil. The *t* test was used to determine the effects of N on efflux and cumulative emission of CO_2_ from biochar, abundance and structure of soil microbial community and DOC derived from biochar. Pearson correlation analysis was carried out if data were normally distributed, otherwise Spearman correlation was adopted. The above statistical analyses were performed with SPSS 16.0 for Windows (SPSS Inc., Chicago, IL, USA). Differences at the *P* < 0.05 level were considered significant. Principal component analysis (PCA) using individual PLFAs concentration at different incubation time was performed to compare the temporal variation of structure of soil microbial community in BC0 and BCN treatments by CANOCO software (Microcomputer Power Inc., Ithaca, NY, USA). Variables (each PLFAs concentration at each incubation time in both treatments) were centered and normalized. The data were compressed into two new independent variables, also known as principal components (PC1 and PC2), which were orthogonal to each other. Following that, the *t* test was carried out to examine the effect of N amendment on PC1 and PC2.

## Results and Discussion

### Biochar decomposition

**Fig 1:**
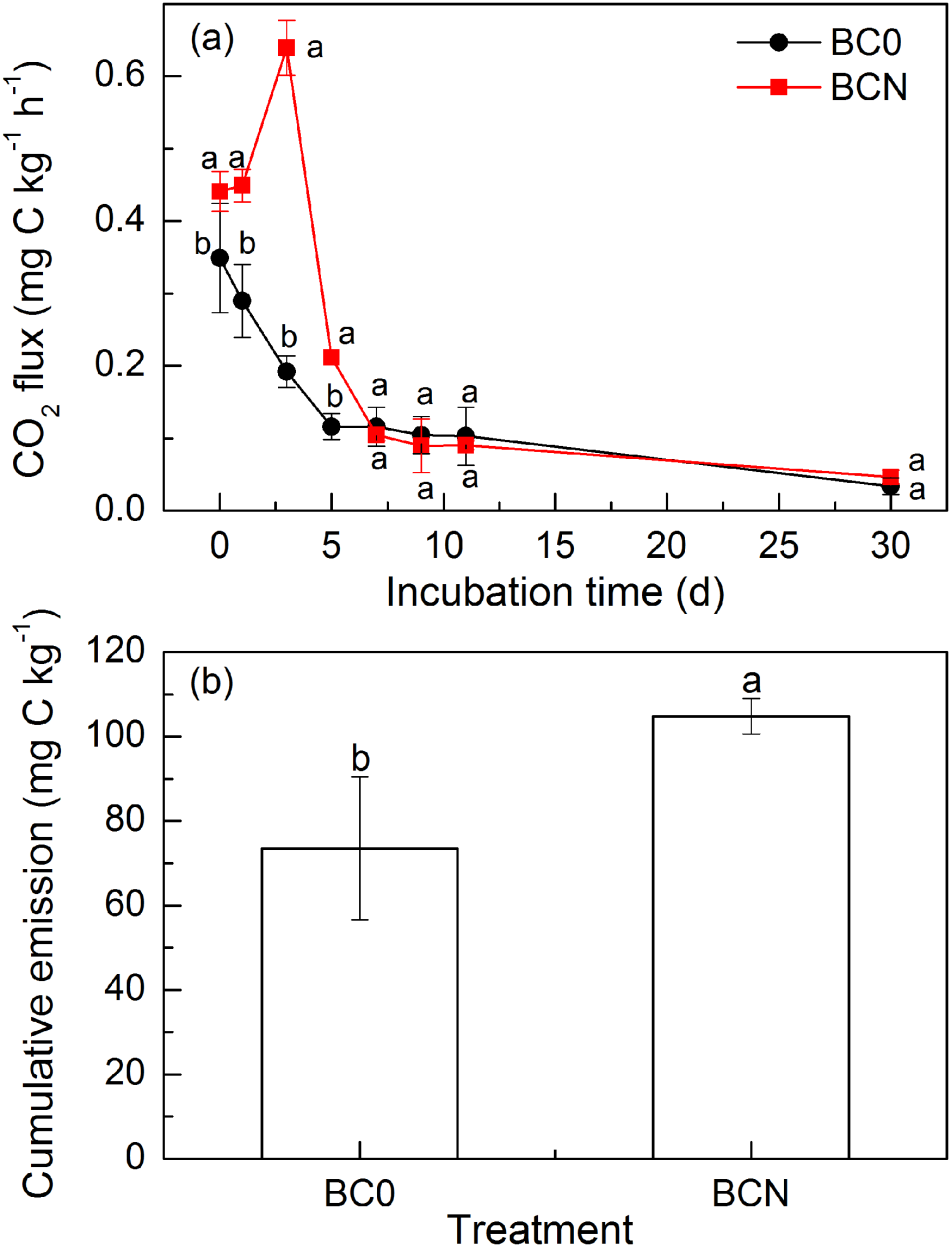
Flux (a) and cumulative emission (b) of carbon dioxide (CO_2_) derived from biochar in soils amended with biochar alone (BC0) and biochar plus nitrogen (BCN) during 30 days of incubation. Different letters denote significant differences at the same incubation time at *P* < 0.05. Vertical bars denote standard errors of the mean (*n* = 3).

Biochar-derived CO_2_ flux peaked at 0.5 h and on day 3 in BC0 and BCN treatments, respectively, and then sharply decreased until day 7 followed by slower decrease until day 30 (Fig 1a). Compared with BC0 treatment, CO_2_ fluxes from biochar were significantly higher in the first 5 days of incubation (*P* < 0.05) but not from day 7 to 30 in BCN treatment. Cumulative CO_2_ emission from biochar was significantly higher in BCN treatment than that in BC0 treatments (*P* < 0.05), with an increase of 42.5% (Fig 1b). The proportion of decomposed biochar C was estimated at 2.30 and 3.28% in BC0 and BCN treatments, respectively. Biochar decomposition proportions in this study were higher than 0.14–0.84% for *M*. *gigantues*-derived biochar pyrolysed at 350 and 700°C in a clay-loam soil during 87 days of incubation [18] and 1.4–1.9% for biochar produced from barley root at 225 and 300°C in a sandy-loam soil during 30 days of incubation [29], but lower than 15.5% for biochar produced from wheat straw at 400°C in the inceptisol during the 117-day rice growth season [30]. Overall, biochar decomposition rates in this study (0.0767–0.1093% biochar C mineralized d^−1^) were in the upper end of previously reported range of 0.0000–0.0479% biochar C mineralized d^−1^ summarized in Ameloot et al. [14] and there were three possible reasons for that. First, the content of hot water extractable DOC (1.75% of biochar C, Table 1) was higher than the reported ranges of 0.03–1.19% [18], and was expected to contribute to the decomposition of biochar because DOC compared with recalcitrant OC was more easily available substrates for and utilized by microorganisms like G^−^bacteria according to previous findings [31–34]. Second, the high mineral contents in biochar as indicated by ash content (Table 1) could stimulate biochar decomposition because minerals might cause defects in aromatic structures, reduce cross links between layers and lower the stability of an overall structure dominated by C links [35]. Third, the sandy-loam texture of the test soil could promote biochar decomposition compared to clayey soils since clay-bound biochar particles might be less available as a C source for microorganisms, concomitantly increasing resistance to biodegradation [36]. More types of biochar and soil should be involved in the study of biochar stability in the future to acquire sufficient and better understanding. Although biochar decomposition rate was reported to be negatively correlated with incubation duration, the initial biochar decomposition in soils seems to be an important aspect when assessing the C sequestration potential [14]. In addition, short-term studies are useful to supply valuable information. However, long-term study is necessary to better understand biochar stability.

### Effect of N amendment on biochar decomposition

**Fig 2:**
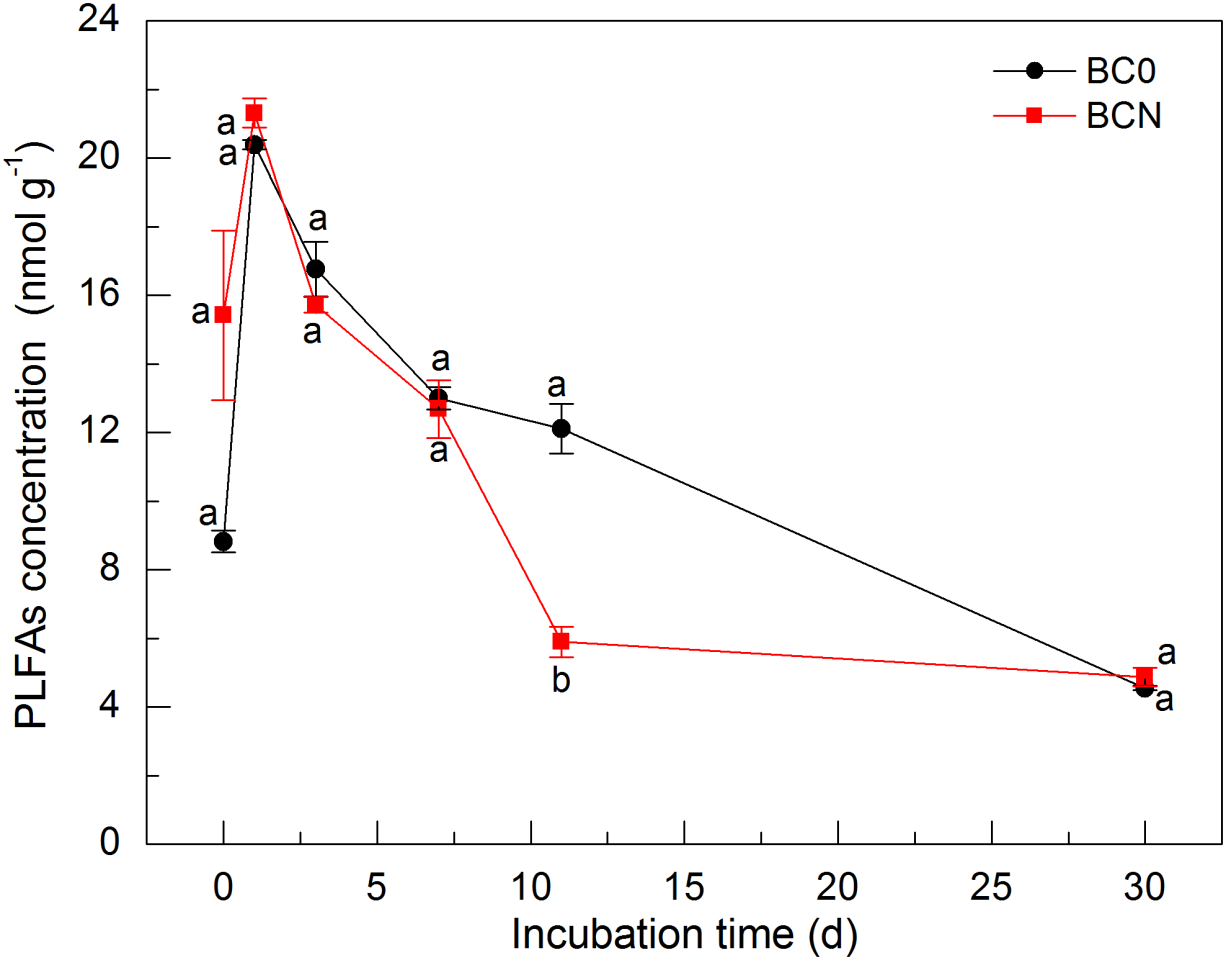
Concentration of phospholipid fatty acids (PLFAs) in soils amended with biochar alone (BC0) and biochar plus nitrogen (BCN) during 30 days of incubation. Different letters denote significant differences at the same incubation time at *P* < 0.05. Vertical bars denote standard errors of the mean (*n* = 3).

**Fig 3:**
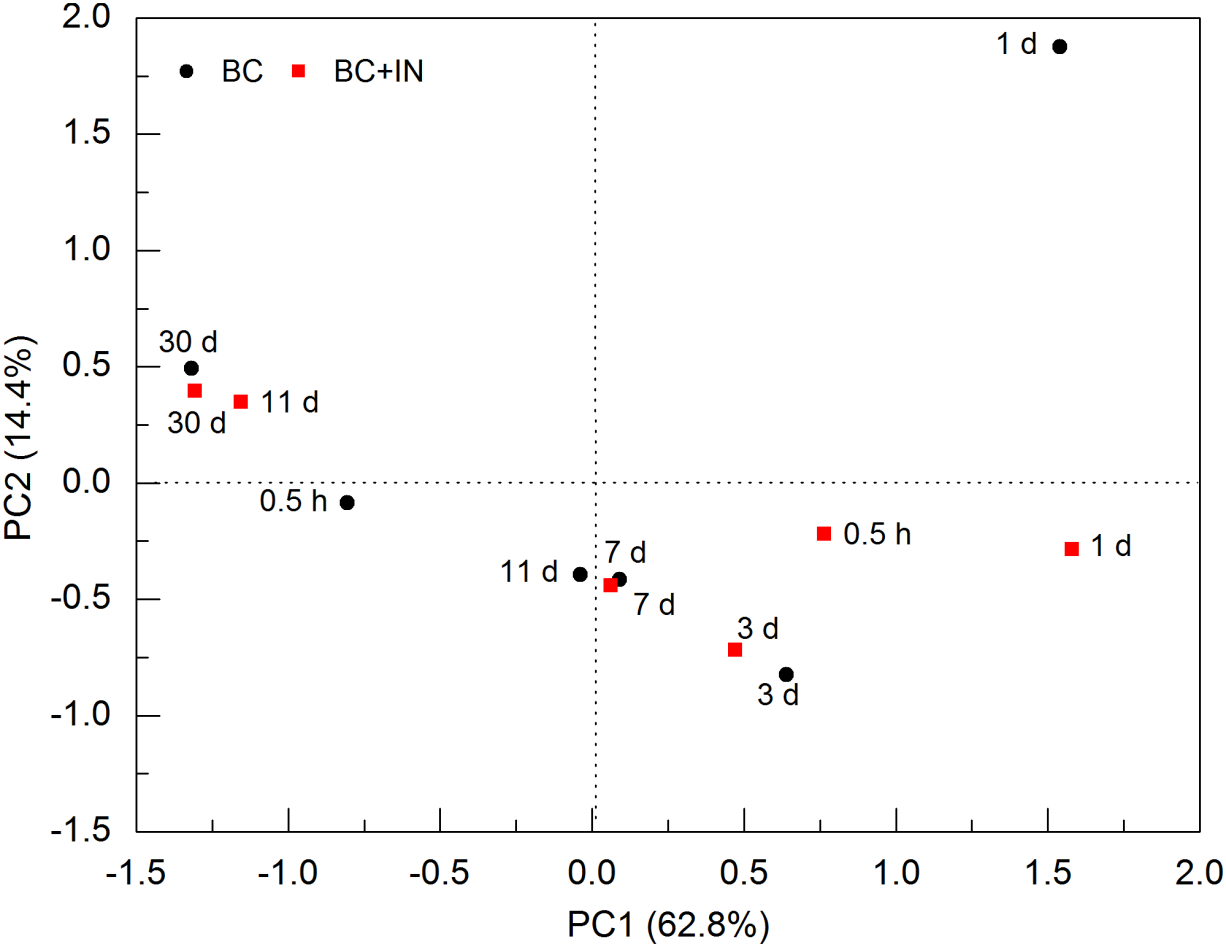
Principal component analysis (PCA) ordination based on the community structure of microorganisms in soils amended with biochar alone (BC0) and biochar plus nitrogen (BCN) at different incubation time. Numbers denote the incubation time while those in the parentheses indicate the percent of variation explained by each axis.

**Fig 4:**
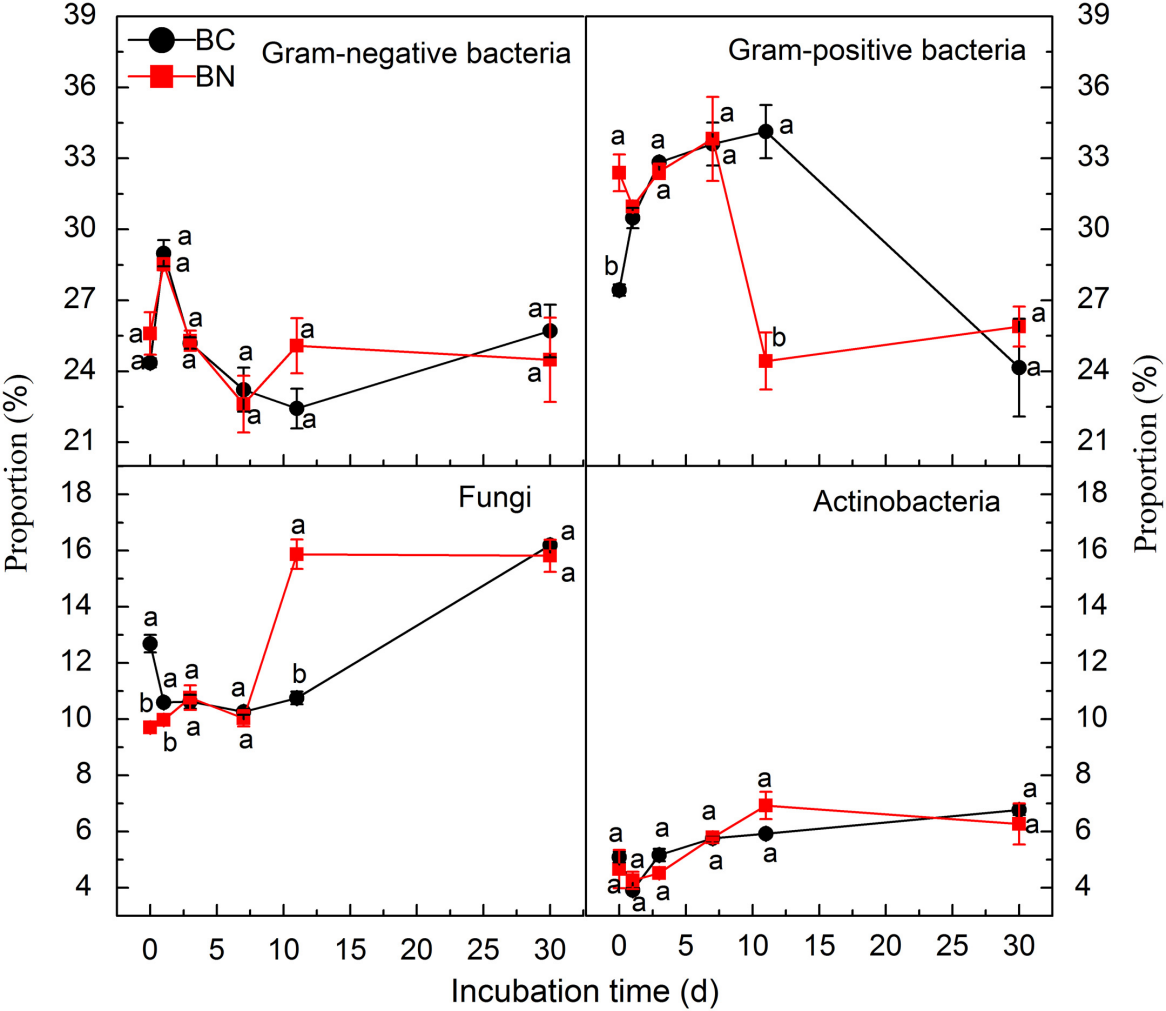
Proportion of different microbial groups as estimated by phospholipid fatty acids (PLFAs) in soils amended with biochar alone (BC0) and biochar plus nitrogen (BCN) during 30 days of incubation. Different letters denote significant differences at the same incubation time at *P* < 0.05. Vertical bars denote standard errors of the mean (*n* = 3). Note that the scales in the upper panels are different from that in the lower panels.

As mentioned above, N amendment substantially stimulated the decomposition of biochar C, as observed for the degradation of native SOC that increased with the application rate of N fertilizer in a Wisconsin upland field [37]. We found that N amendment increased PLFAs concentration at 0.5 h and in contrast, significantly decreased it on day 11 (*P* < 0.05) in soils amended with biochar (Fig 2). Interestingly, no apparent difference was observed on the other days of incubation. Ding et al. [10] also measured a 33.0% increase of microbial biomass when ammonium (NH_4_^+^), rather than nitrate (NO_3_^−^), was added to a similar soil after 25 days of incubation. These results suggested that N could be limited for microbial proliferation in test soil [38]. N amendment did not lead to alterations in the PLFAs profiles over 30 days of incubation except at 0.5 h and on day 11 (Fig 3), indicating that N amendment dramatically changed soil microbial community structure only at 0.5 h and on day 11. Similar phenomenon was also observed by Thirukkumaran and Parkinson [39]. Further analysis showed that N amendment significantly increased the proportion of G^+^ bacteria (*P* < 0.05) but decreased that of fungi at 0.5 h (*P* < 0.05), while an opposite result was observed on day 11 due to the decreased abundance of G^+^ bacteria (Fig 4). Previous studies have shown that N amendment suppressed the growth of fungi in grassland and forestland soils [40–41]. However, there is conflicting evidence in the literature about the effect of N addition on bacterial species. For instance, Rinnan et al. [42] and Denef et al. [43] detected increases in the relative abundance of G^+^ bacteria with N fertilization in tundra soil and temperate grassland soil. In contrast, Billings and Ziegler [7] reported that N fertilization enhanced G^−^bacteria whereas reduced the proportion of G^+^ bacteria. As it was suggested that the relative abundance of various microbial groups in soils might affect their responses to additive substrates [23], it was speculated that the greater proliferation of G^+^ bacteria at the beginning of incubation induced by N amendment was primarily due to their initial higher abundance in test soil prior to N addition, although other factors, such as soil pH and C/N ratio might also play a role [44–46]. Besides microbial community structure, N could also influence the activity of soil microbial community as characterized by enzyme activity. Previous studies showed that N amendment could increase the activity of cellulose-decomposing enzymes, but lowered the activity of lignin-degrading enzymes such as phenol oxidases and perioxidases [47–50].

Correlation analysis indicated that biochar-derived CO_2_ efflux was significantly (*P* < 0.05) correlated with PLFAs concentration in BCN treatment (Table 2), indicating that soil microbial abundance was the key factor controlling biochar decomposition. Significant increase was found for the proportion of G^+^ bacteria rather than that of fungi and actinobacteria as affected by N amendment at the early stage of incubation (Fig 4). Because the promotion of biochar-derived CO_2_ efflux by N amendment mainly occurred during the first 5 days (Fig 1a), it is speculated that the enhanced decomposition of biochar by N amendment was primarily due to promotion of G^+^ bacteria. G^+^ bacteria are known to decompose a range of organic materials, including relatively recalcitrant substances such as lignin and aromatic/alkenes-C that are found in high concentrations in biochar by producing exoenzymes [51–54]. While available inorganic N in soils is required for G^+^ bacteria to invest in extracellular enzymes [55]. Furthermore, studies have demonstrated that G^+^ bacteria not only could directly utilize biochar C but also were the primary utilizers of biochar derived from *Pinus ponderosa* [56, 8]. In contrast, fungi were able to degrade and metabolize the unpyrolysed pine wood rather than pyrolysed biochar probably due to the unsuitability of biochar as a substrate for fungi and/or due to no colonization of fungi on wood-derived biochar, apart from a small number of cracks within the biochar [57]. Interestingly, no significant correlation was found between biochar-derived CO_2_ efflux and PLFAs concentration in BC0 treatment (Table 2). It is likely that the decomposition of labile OC was closely correlated with the activity rather than biomass of microbes especially G^−^bacteria since part of microbes remained dormant [58–60]. Another possibility is that the activity of increased G^+^ bacteria was partially, if not fully inhibited by the deficiency of available inorganic N in BC0 treatment [55].

**Table 2:** Relationship between CO_2_ flux and the concentration of phospholipid fatty acids (PLFAs) or dissolved organic carbon (DOC) in soils amended with biochar alone (BC0) and biochar plus nitrogen (BCN) during 30 days of incubation.

Treatment	PLFAs		DOC	
	*R* value	*P* value	*R* value	*P* value
**BC0**	0.45	0.452	0.58	0.304
**BCN**	0.88	0.049	0.81	0.097

**Fig 5:**
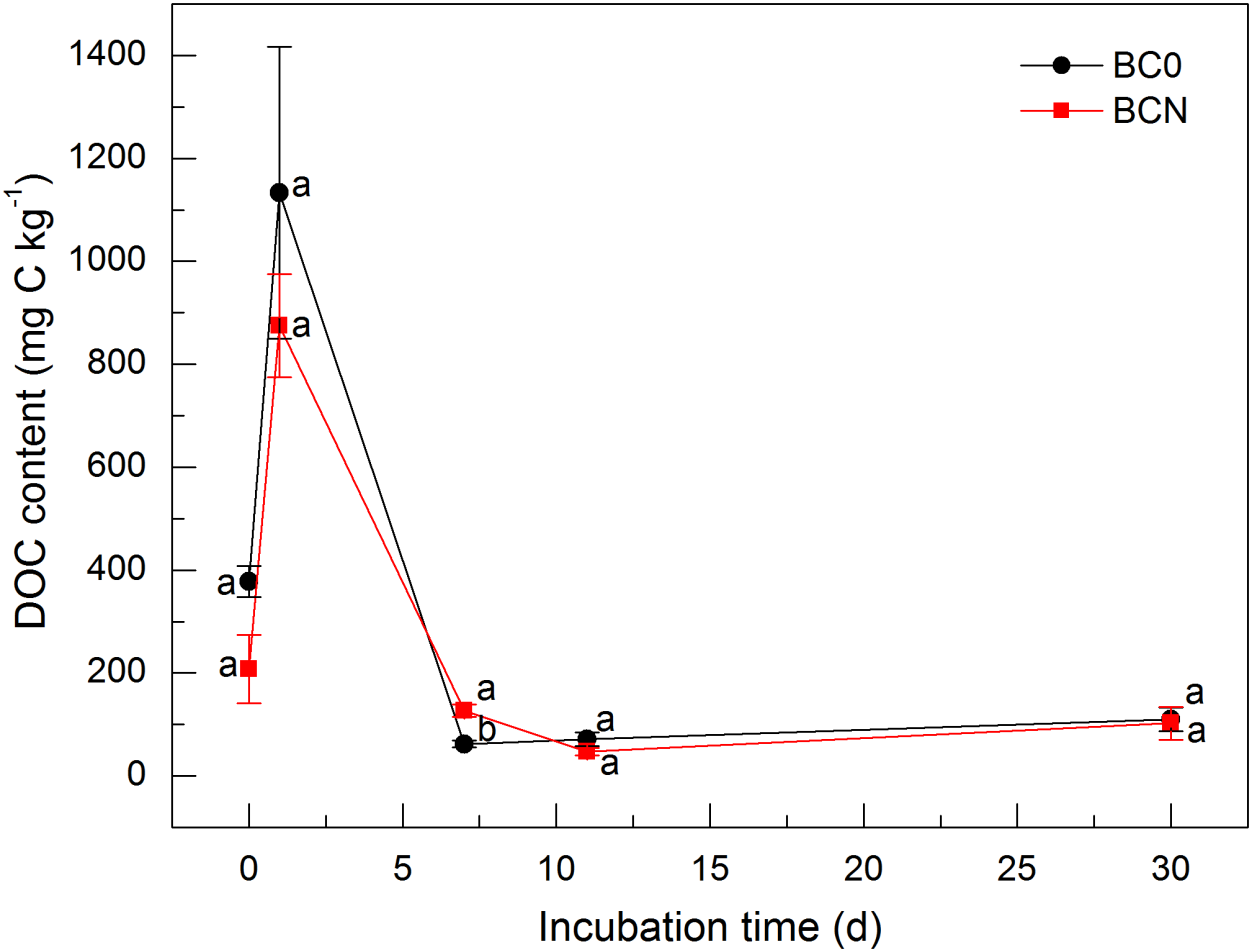
Content of dissolved organic carbon (DOC) derived from biochar in soils amended with biochar alone (BC0) and biochar plus nitrogen (BCN) during 30 days of incubation. Different letters denote significant differences at the same incubation time at *P* < 0.05. Vertical bars denote standard errors of the mean (*n* = 3).

During the incubation, biochar-derived DOC generally had no significant differences between BC0 and BCN treatments (*P* > 0.05; Fig 5), suggesting that N amendment primarily stimulated the decomposition of recalcitrant OC rather than DOC in biochar. The content of biochar-derived DOC peaked on day 1, being 874.9 and 1132.9 mg C kg^–1^, then rapidly decreased to 127.2 and 61.7 mg C kg^–1^ on day 7 in BC0 and BCN treatments, respectively, and finally maintained rather constant until day 30. The rapid decrease of DOC derived from biochar at the early stage of incubation was also reported by Bruun et al. [29]. This was induced by the preferential utilization of labile OC compared with relatively recalcitrant OC by microbes [12, 61]. The initial stage of fast mineralization has been reported to last between 2 and 60 days [6, 61], during which 2–20% of the biochar C can be mineralized [14].

Correlation analysis indicated that the correlation between CO_2_ efflux and DOC content derived from biochar was not significant in both treatments (*P* > 0.05), indicating that there were other factors influencing biochar decomposition besides labile OC contained in biochar. The proportion of decomposed biochar C was higher than the proportion of DOC in biochar C (1.75%) as well as that of O/N-alkyl C (1.28%), which is thought to be labile C functional group [62], indicating that part of relatively recalcitrant OC might also be decomposed during the incubation. In extracts of citrus wood biochar with a dichloromethane and methanol mixture (the volume ratio of 95:5), a wide array of relatively low molecular weight organic compounds were identified, including n-alkanoic acids, hydroxyl and acetoxy acids, benzoic acids, diols, triols and phenols [63]. Kramer et al. [64] demonstrated that DOC also contained recalcitrant compounds with long turnover time such as aromatic acids. Thus, the biochar-derived DOC remaining in soils might be relatively difficult to be used by microorganisms. More importantly, it is also likely that previously non-extractable biochar C was slowly released during the abiotic and microbial mineralization and replenished DOC, as previously reported by Zimmerman and Gao [65].

Using the ^13^C NMR technique, Hilscher and Knicker [66] found that after 20 months of incubation, the alkyl C in rye grass biochar decreased only by 5%, while up to 26–40% of the initial aryl C amount was mineralized or converted to other functional groups and in contrast, the proportion of carboxyl/carbonyl C increased to ~15% in the remaining biochar from ~10% in the initial biochar. The decomposition rate of aryl ^14^C in lignin amounted to 0.079–0.167% aryl C mineralized d^−1^ during the 41 days of incubation [67]. These results indicated that aryl C in biochar, which accounted for 91.1% of biochar C in this study, could also be decomposed. Cheng and Kuzyakov [68] observed that root exudates-induced priming effect on the decomposition of recalcitrant SOC was greater than on mineralization of labile OC in residues. Thus, we consider that N amendment stimulated proliferation of G^+^ bacteria in test soil, which in turn accentuated decomposition of aryl C in biochar and reduced the stability of biochar C. Further study is required to understand the dynamic variation of functional groups in biochar as affected by N amendment.

## Conclusions

Nitrogen amendment can enhance the decomposition of maize straw-derived biochar in the test sandy loam soil especially at the early stage of decomposition. Microorganisms are N-limited and N amendment can effectively enhance the proportion of G^+^ bacteria immediately probably due to its initial dominance in test soil, which probably accelerated degradation of relatively recalcitrant biochar C. Therefore, N amendment will enhance the decomposition of maize straw-derived biochar probably due to the promotion of G^+^ bacteria in test soil.

## References

[1] Lehmann J. Bio-energy in the black. Front Ecol Environ. 2007; 5: 381–387.

[2] Glaser B, Lehmann J, Zech W. Ameliorating physical and chemical properties of highly weathered soils in the tropics with charcoal—a review. Biol Fert Soils. 2002; 35: 219–230.

[3] Jeffery S, Verheijen FGA, van der Velde M, Bastos AC. A quantitative review of the effects of biochar application to soils on crop productivity using meta-analysis. Agr Ecosyst Environ. 2011; 144: 175–187.

[4] Baldock JA, Smernik RJ. Chemical composition and bioavailability of thermally altered Pinus resinosa (Red pine) wood. Org Geochem. 2002; 33: 1093–1109.

[5] Zimmerman AR. Abiotic and microbial oxidation of laboratory-produced black carbon (biochar). Environ Sci Technol. 2010; 44: 1295–1301. 10.1021/es903140c 20085259

[6] Steinbeiss S, Gleixner G, Antonietti M. Effect of biochar amendment on soil carbon balance and soil microbial activity. Soil Biol Biochem. 2009; 41: 1301–1310.

[7] Billings SA, Ziegler SE. Altered patterns of soil carbon substrate usage and heterotrophic respiration in a pine forest with elevated CO_2_ and N fertilization. Global Change Biol. 2008; 14: 1025–1036.

[8] Santos F, Torn MS, Bird JA. Biological degradation of pyrogenic organic matter in temperate forest soils. Soil Biol Biochem. 2012; 51: 115–124.

[9] Schulz H, Glaser B. Effects of biochar compared to organic and inorganic fertilizers on soil quality and plant growth in a greenhouse experiment. J Plant Nutr Soil Sc. 2012; 175: 410–422.

[10] Ding WX, Yu HY, Cai ZC, Han FX, Xu ZH. Responses of soil respiration to N fertilization in a loamy soil under maize cultivation. Geoderma. 2010; 155: 381–389.

[11] Uchimiya M, Ohno T, He Z. Pyrolysis temperature-dependent release of dissolved organic carbon from plant, manure, and biorefinery wastes. J Anal Appl Pyrol. 2013; 104: 84–94.

[12] Cross A, Sohi SP. The priming potential of biochar products in relation to labile carbon contents and soil organic matter status. Soil Biol Biochem. 2011; 43: 2127–2134.

[13] Spokas KA. Review of the stability of biochar in soils: Predictability of O:C molar ratios. Carbon Manag. 2010; 1: 289–303.

[14] Ameloot N, Graber ER, Verheijen FGA, Deneve S. Interactions between biochar stability and soil organisms: Review and research needs. Eur J Soil Sci. 2013; 64: 379–390.

[15] Watzinger A, Feichtmair S, Kitzler B, Zehetner F, Kloss S, Wimmer B. Soil microbial communities responded to biochar application in temperate soils and slowly metabolized ^13^C-labelled biochar as revealed by ^13^C PLFA analyses: Results from a short-term incubation and pot experiment. Eur J Soil Sci. 2014; 65: 40–51.

[16] USDA (United States Department of Agriculture). Keys to Soil Taxonomy (Soil Conservation Service). Sixth ed. 1994.

[17] Novak JM, Busscher WJ, Laird DL, Ahmedna M, Watts DW, Niandou MAS. Impact of biochar amendment on fertility of a southeastern coastal plain soil. Soil Sci. 2009; 174: 105–112.

[18] Luo Y, Durenkamp M, De Nobili M, Lin Q, Brookes PC. Short term soil priming effects and the mineralisation of biochar following its incorporation to soils of different pH. Soil Biol Biochem. 2011; 43: 2304–2314.

[19] Midwood AJ, Boutton TW. Soil carbonate decomposition by acid has little effect on the δ^13^C of organic matter. Soil Biol Biochem. 1998; 30: 1301–1307.

[20] Lu RK. Methods of Soil and Agrochemical Analysis. Beijing: China Agricultural Science and Technology Press; 2000. (In Chinese)

[21] Chodak M, Khanna P, Beese F. Hot water extractable C and N in relation to microbiological properties of soils under beech forests. Biol Fert Soils. 2003; 39: 123–130.

[22] Solum MS, Pugmire RJ, Grant DM. ^13^C solid-state NMR of Argonne premium coals. Energ Fuel. 1989; 3: 187–193.

[23] Zhang HJ, Ding WX, Yu HY, He XH. Carbon uptake by a microbial community during 30-day treatment with ^13^C-glucose of a sandy loam soil fertilized for 20 years with NPK or compost as determined by a GC-C-IRMS analysis of phospholipid fatty acids. Soil Biol Biochem. 2013; 57: 228–236.

[24] Cai YJ, Ding WX, Luo JF. Nitrous oxide emissions from Chinese maize—wheat rotation systems: A 3-year field measurement. Atmos Environ. 2013; 65: 112–122.

[25] Brant JB, Sulzman EW, Myrold DD. Microbial community utilization of added carbon substrates in response to long-term carbon input manipulation. Soil Biol Biochem. 2006; 38: 2219–2232.

[26] Spring S, Schulze R, Overmann J, Schleifer KH. Identification and characterization of ecologically significant prokaryotes in the sediment of freshwater lakes: Molecular and cultivation studies. FEMS Microbiol Rev. 2000; 24: 573–590. 11077151 10.1111/j.1574-6976.2000.tb00559.x

[27] Amelung W, Brodowski S, Sandhage-Hofmann A, Bol R. Combining biomarker with stable isotope analyses for assessing the transformation and turnover of soil organic matter In: Donald LS, editor. Advances in Agronomy. Burlington: Academic Press; 2008 pp. 155–250.

[28] Balesdent J, Mariotti A. Measurement of soil organic matter turnover using ^13^C natural abundance In: Boutton TW, Yamasaki S, editors. Mass Spectrometry of Soils. New York: Marcel Dekker; 1996 pp. 83–111.

[29] Bruun S, Jensen E, Jensen L. Microbial mineralization and assimilation of black carbon: Dependency on degree of thermal alteration. Org Geochem. 2008; 39: 839–845.

[30] Xie ZB, Xu YP, Liu G, Liu Q, Zhu JG, Tu C. Impact of biochar application on nitrogen nutrition of rice, greenhouse-gas emissions and soil organic carbon dynamics in two paddy soils of China. Plant Soil. 2013; 370: 527–540.

[31] Kuehn KA, Lemke MJ, Suberkropp K, Wetzell RG. Microbial biomass and production associated with decaying leaf litter of the emergent macrophyte *Juncus effuses* . Limnol Oceanogr. 2000; 45: 862–870.

[32] Blagodatskaya EV, Blagodatsky SA, Anderson TH, Kuzyakov Y. Contrasting effects of glucose, living roots and maize straw on microbial growth kinetics and substrate availability in soil. Eur J Soil Sci. 2009; 60: 186–197.

[33] Kuzyakov Y, Cheng W. Photosynthesis controls of CO_2_ efflux from maize rhizosphere. Plant Soil. 2004; 263: 85–99.

[34] Li LJ, Zeng DH, Yu ZA, Fan ZP, Mao R. Soil microbial properties under N and P additions in a semi-arid, sandy grassland. Biol Fert Soils. 2010; 46: 653–658.

[35] Nguyen BT, Lehmann J. Black carbon decomposition under varying water regimes. Org Geochem. 2009; 40: 846–853.

[36] Chenu C, Plante AF. Clay-sized organo-mineral complexes in a cultivation chronosequence: Revisiting the concept of the ‘primary organo-mineral complex’. Eur J Soil Sci. 2006; 57: 596–607.

[37] Vanotti MB, Bundy LG, Peterson AE. Nitrogen fertilizer and legume-cereal rotation effects on soil productivity and organic matter dynamics in Wisconsin In: Paul EA, Paustian KH, Elliott ET, Cole CV, editors. Soil organic matter in temperate agroecosystems: Long-term experiments in North America. Florida: CRC Press; 1996 pp. 105–119.

[38] Compton JE, Watrud LS, Porteous LA, DeGrood S. Response of soil microbial biomass and community composition to chronic nitrogen additions at Harvard forest. Forest Ecol Manag. 2004; 196: 143–158.

[39] Thirukkumaran C, Parkinson D. Microbial respiration, biomass, metabolic quotient and litter decomposition in a lodgepole pine forest floor amended with nitrogen and phosphorous fertilizers. Soil Biol Biochem. 2000; 32: 59–66.

[40] De Vries FT, Bloem J, van Eekeren N, Brussaard L, Hoffland E. Fungal biomass in pastures increases with age and reduced N input. Soil Biol Biochem. 2007; 39: 1620–1630.

[41] Demoling F, Nilsson LO, Bääth E. Bacterial and fungal response to nitrogen fertilization in three coniferous forest soils. Soil Biol Biochem. 2008; 40: 370–379.

[42] Rinnan R, Michelsen A, Bååth E, Jonasson S. Fifteen years of climate change manipulations alter soil microbial communities in a subarctic heath ecosystem. Global Change Biol. 2007; 13: 28–39.

[43] Denef K, Roobroeck D, Manimel Wadu MCW, Lootens P, Boeckx P. Microbial community composition and rhizodeposit-carbon assimilation in differently managed temperate grassland soils. Soil Biol Biochem. 2009; 41: 144–153.

[44] Gul S, Whalen JK, Thomas BW, Sachdeva V, Deng HY. Physico-chemical properties and microbial responses in biochar-amended soils: Mechanisms and future directions. Agr Ecosyst Environ. 2015; 206: 46–59.

[45] Ai C, Liang G, Sun J, He P, Tang S, Yang S, et al The alleviation of acid soil stress in rice by inorganic or organic ameliorants is associated with changes in soil enzyme activity and microbial community composition. Biol Fert Soils. 2015; 51: 465–477.

[46] Tscherko D, Hammesfahr U, Marx M, Kandeler E. Shifts in rhizosphere microbial communities and enzyme activity of *Poa alpine* across an alpine chronosequence. Soil Biol Biochem. 2004; 36: 1685–1698.

[47] Carreiro MM, Sinsabaugh RL, Repert DA, Parkhurst DF. Microbial enzyme shifts explain litter decay responses to simulated nitrogen deposition. Ecology. 2000; 81: 2359–2365.

[48] Frey SD, Knorr M, Parrent JL, Simpson RT. Chronic nitrogen enrichment affects the structure and function of the soil microbial community in temperate hardwood and pine forests. Forest Ecol Manag. 2004; 196: 159–171.

[49] Keeler BL, Hobbie SE, Kellogg LE. Effects of long-term nitrogen addition on microbial enzyme activity in eight forested and grassland sites: Implications for litter and soil organic matter decomposition. Ecosystems. 2009; 12: 1–15.

[50] Min K, Kang H, Lee D. Effects of ammonium and nitrate additions on carbon mineralization in wetland soils. Soil Biol Biochem. 2011; 43: 2461–2469.

[51] McMahon SK, Williams MA, Bottomley PJ, Myrold DD. Dynamics of microbial communities during decomposition of carbon-13 labeled ryegrass fractions in soil. Soil Sci Soc Am J. 2005; 69: 1238–1247.

[52] Bird JA, Herman DJ, Firestone MK. Rhizosphere priming of soil organic matter by bacterial groups in a grassland soil. Soil Biol Biochem. 2011; 43: 718–725.

[53] Chapman SK, Koch GW. What type of diversity yields synergy during mixed litter decomposition in a natural forest ecosystem? Plant Soil. 2007; 299: 153–162.

[54] Biasi C, Rusalimova O, Meyer H, Kaiser C, Wanek W, Barsukov P, et al Temperature-dependent shift from labile to recalcitrant carbon sources of arctic heterotrophs. Rapid Commun Mass Sp. 2005; 19: 1401–1408. 10.1002/rcm.1911 15880633

[55] Treseder KK, Kivlin SN, Hawkes CV. Evolutionary trade-offs among decomposers determine responses to nitrogen enrichment. Ecol Lett. 2011; 14: 933–938. 10.1111/j.1461-0248.2011.01650.x 21749597

[56] Farrell M, Kuhn TK, Macdonald LM, Maddern TM, Murphy DV, Hall PA, et al Microbial utilisation of biochar-derived carbon. Sci Total Environ. 2013; 465: 288–297. 10.1016/j.scitotenv.2013.03.090 23623696

[57] Ascough PL, Sturrock CJ, Bird MI. Investigation of growth responses in saprophytic fungi to charred biomass. Isot Environ Health Stud. 2010; 46: 64–77. 10.1080/10256010903388436 20229385

[58] Butler JL, Williams MA, Bottomley PJ, Myrold DD. Microbial community dynamics associated with rhizosphere carbon flow. Appl Environ Microbiol. 2003; 69: 6793–6800. 14602642 10.1128/AEM.69.11.6793-6800.2003 PMC262268

[59] Michelsen A, Andersson M, Jensen M, Kjøller A, Menassie Gashew M. Carbon stocks, soil respiration and microbial biomass in fire-prone tropical grassland, woodland and forest ecosystems. Soil Biol Biochem. 2004; 36: 1707–1717.

[60] Potthoff M, Dyckmans J, Flessa H, Muhs M, Beese S, Joergensen RG. Dynamics of maize (*Zea mays* L.) leaf straw mineralization as affected by the presence of soil and the availability of nitrogen. Soil Biol Biochem. 2005; 37: 1259–1266.

[61] Smith JL, Collins HP, Bailey VL. The effect of young biochar on soil respiration. Soil Biol Biochem. 2010; 42: 2345–2347.

[62] Baldock JA, Oades JM, Waters AG, Peng X, Vassallo AM, Wilson MA. Aspects of the chemical structure of soil organic materials as revealed by solid-state ^13^C NMR spectroscopy. Biogeochemistry. 1992; 16: 1–42.

[63] Graber ER, Harel YM, Kolton M, Cytryn E, Silber A, David DR. Biochar impact on development and productivity of pepper and tomato grown in fertigated soilless media. Plant Soil. 2010; 337: 481–496.

[64] Kramer MG, Sanderman J, Chadwick OA, Chorover J, Vitousek PM. Long-term carbon storage through retention of dissolved aromatic acids by reactive particles in soil. Global Change Biol. 2012; 18: 2594–2605.

[65] Zimmerman AR, Gao B. The stability of biochar in the environment In: Ladygina N, Rineau F, editors. Biochar and Soil Biota. Boca Raton: CRC press; 2013 pp. 1–40.

[66] Hilscher A, Knicker H. Carbon and nitrogen degradation on molecular scale of grass-derived pyrogenic organic material during 28 months of incubation in soil. Soil Biol Biochem. 2011; 43: 261–270.

[67] Hackett WF, Connors WJ, Kirk TK, Zeikus JG. Microbial decomposition of synthetic ^14^C-labeled lignins in nature: Lignin biodegradation in a variety of natural materials. Appl Environ Microbiol. 1977; 33: 43–51. 16345189 10.1128/aem.33.1.43-51.1977 PMC170572

[68] Cheng WX, Kuzyakov Y. Root effects on soil organic matter decomposition In: Zobel RW, Wright SF, editors. Roots and Soil Management: Interactions between Roots and the Soil. Wisconsin: ASA-SSSA; 2005 pp. 119–143.

